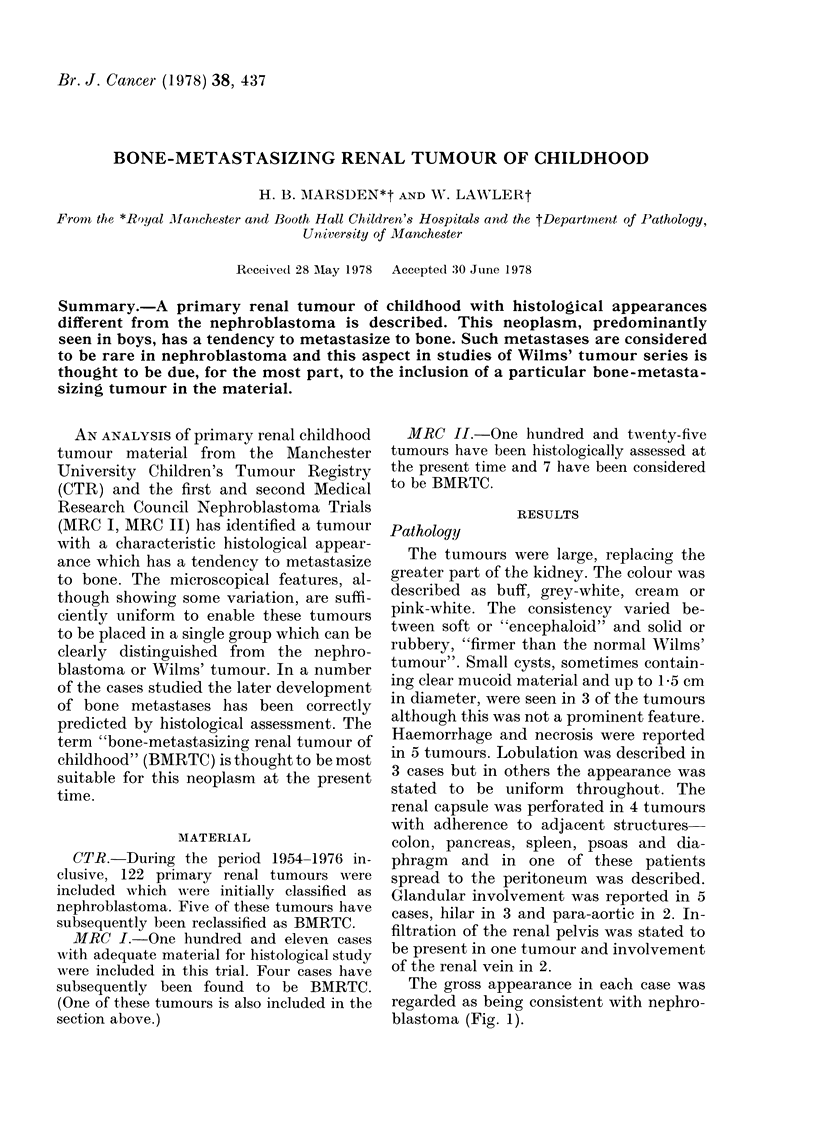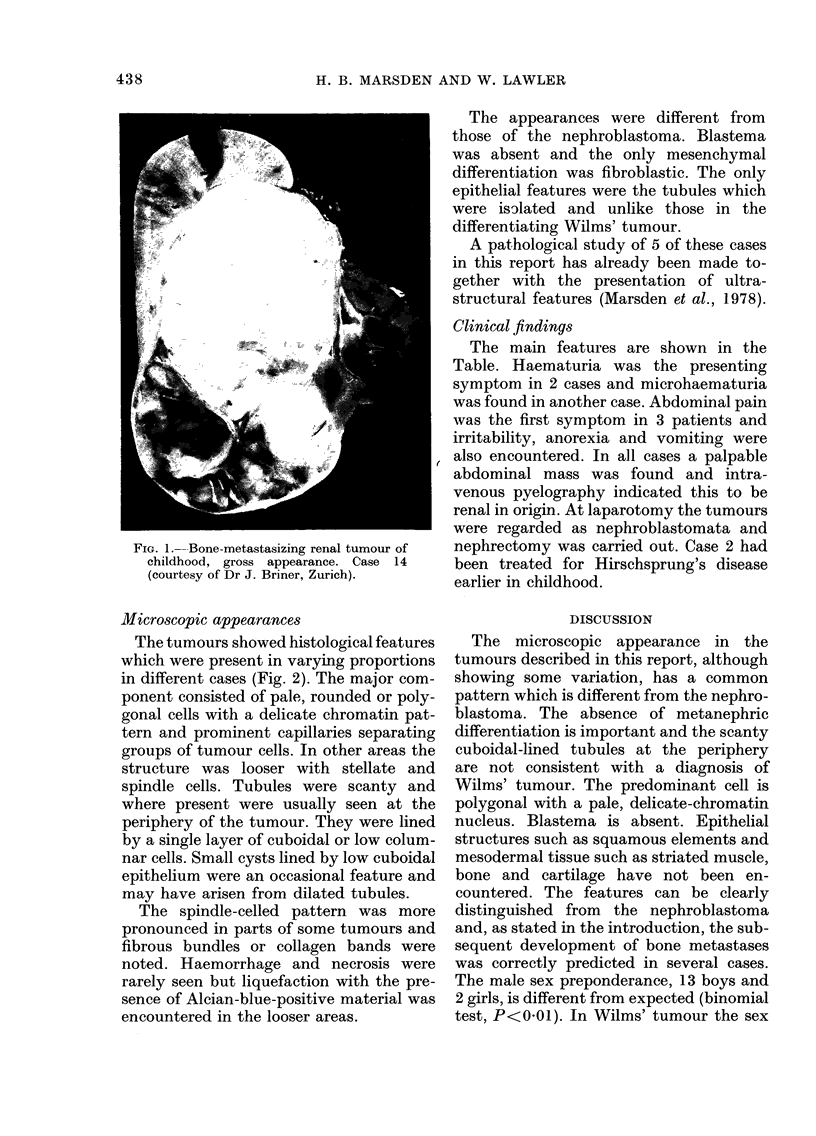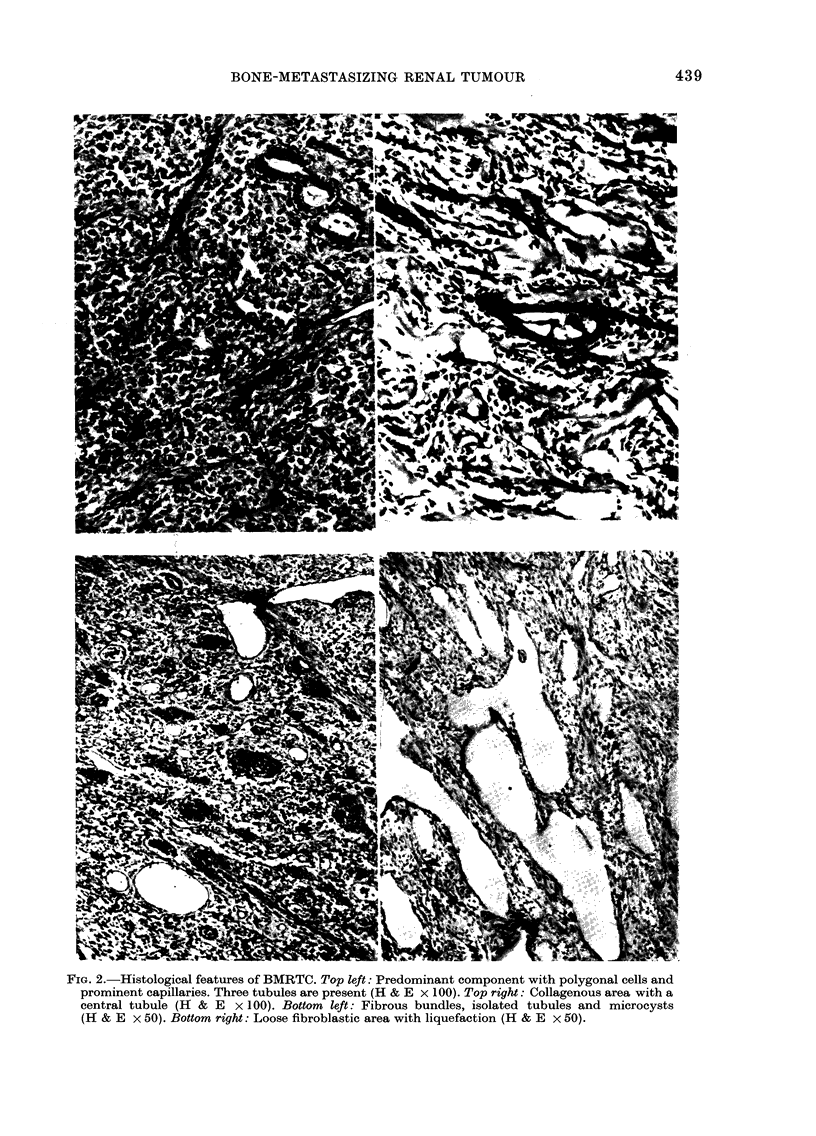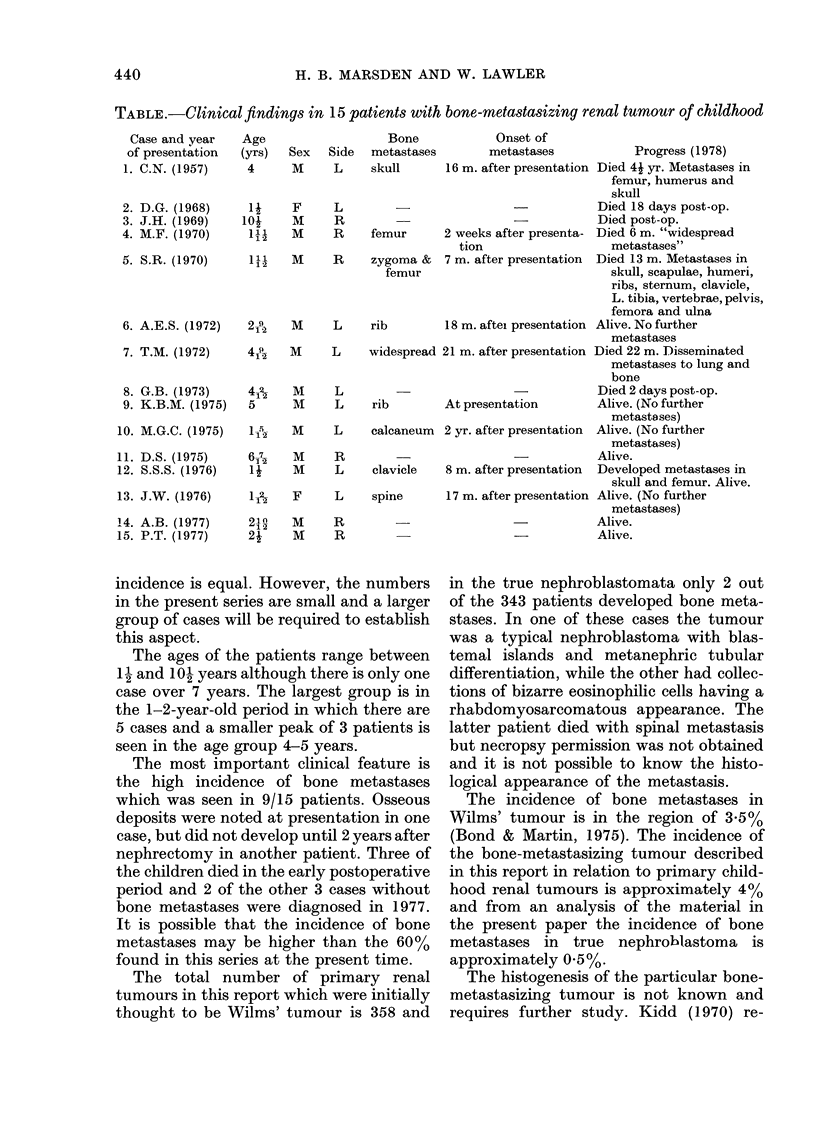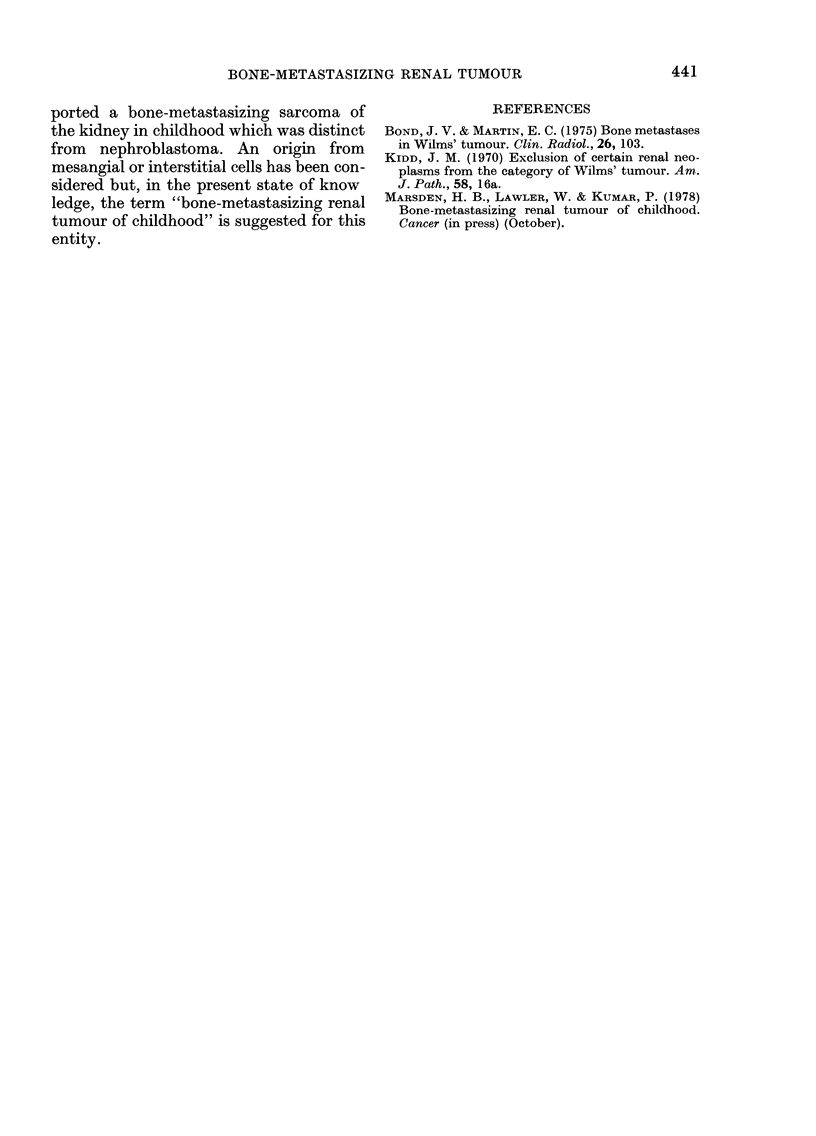# Bone-metastasizing renal tumour of childhood.

**DOI:** 10.1038/bjc.1978.226

**Published:** 1978-09

**Authors:** H. B. Marsden, W. Lawler

## Abstract

**Images:**


*Br. J. Cancer* (1978) **38**, 437

### BONE-METASTASIZING RENAL TUMOUR OF CHILDHOOD

H. B. MARSDEN*† AND W. LAWLER†

*From the *Royal Manchester and Booth Hall Children's Hospitals and the †Department of Pathology, University of Manchester*

Received 28 May 1978 Accepted 30 June 1978

**Summary.—A primary renal tumour of childhood with histological appearances different from the nephroblastoma is described. This neoplasm, predominantly seen in boys, has a tendency to metastasize to bone. Such metastases are considered to be rare in nephroblastoma and this aspect in studies of Wilms' tumour series is thought to be due, for the most part, to the inclusion of a particular bone-metastasizing tumour in the material.**

AN ANALYSIS of primary renal childhood tumour material from the Manchester University Children's Tumour Registry (CTR) and the first and second Medical Research Council Nephroblastoma Trials (MRC I, MRC II) has identified a tumour with a characteristic histological appearance which has a tendency to metastasize to bone. The microscopical features, although showing some variation, are sufficiently uniform to enable these tumours to be placed in a single group which can be clearly distinguished from the nephroblastoma or Wilms' tumour. In a number of the cases studied the later development of bone metastases has been correctly predicted by histological assessment. The term "bone-metastasizing renal tumour of childhood" (BMRTC) is thought to be most suitable for this neoplasm at the present time.

### MATERIAL

*CTR.*—During the period 1954–1976 inclusive, 122 primary renal tumours were included which were initially classified as nephroblastoma. Five of these tumours have subsequently been reclassified as BMRTC.

*MRC I.*—One hundred and eleven cases with adequate material for histological study were included in this trial. Four cases have subsequently been found to be BMRTC. (One of these tumours is also included in the section above.)

*MRC II.*—One hundred and twenty-five tumours have been histologically assessed at the present time and 7 have been considered to be BMRTC.

### RESULTS

### *Pathology*

The tumours were large, replacing the greater part of the kidney. The colour was described as buff, grey-white, cream or pink-white. The consistency varied between soft or "encephaloid" and solid or rubbery, "firmer than the normal Wilms' tumour". Small cysts, sometimes containing clear mucoid material and up to 1·5 cm in diameter, were seen in 3 of the tumours although this was not a prominent feature. Haemorrhage and necrosis were reported in 5 tumours. Lobulation was described in 3 cases but in others the appearance was stated to be uniform throughout. The renal capsule was perforated in 4 tumours with adherence to adjacent structures—colon, pancreas, spleen, psoas and diaphragm and in one of these patients spread to the peritoneum was described. Glandular involvement was reported in 5 cases, hilar in 3 and para-aortic in 2. Infiltration of the renal pelvis was stated to be present in one tumour and involvement of the renal vein in 2.

The gross appearance in each case was regarded as being consistent with nephroblastoma (Fig. 1).

FIG. 1.—Bone-metastasizing renal tumour of childhood, gross appearance. Case 14 (courtesy of Dr J. Briner, Zurich).

*Microscopic appearances*

The tumours showed histological features which were present in varying proportions in different cases (Fig. 2). The major component consisted of pale, rounded or polygonal cells with a delicate chromatin pattern and prominent capillaries separating groups of tumour cells. In other areas the structure was looser with stellate and spindle cells. Tubules were scanty and where present were usually seen at the periphery of the tumour. They were lined by a single layer of cuboidal or low columnar cells. Small cysts lined by low cuboidal epithelium were an occasional feature and may have arisen from dilated tubules.

The spindle-celled pattern was more pronounced in parts of some tumours and fibrous bundles or collagen bands were noted. Haemorrhage and necrosis were rarely seen but liquefaction with the presence of Alcian-blue-positive material was encountered in the looser areas.

The appearances were different from those of the nephroblastoma. Blastema was absent and the only mesenchymal differentiation was fibroblastic. The only epithelial features were the tubules which were isolated and unlike those in the differentiating Wilms' tumour.

A pathological study of 5 of these cases in this report has already been made together with the presentation of ultrastructural features (Marsden *et al.*, 1978).

*Clinical findings*

The main features are shown in the Table. Haematuria was the presenting symptom in 2 cases and microhaematuria was found in another case. Abdominal pain was the first symptom in 3 patients and irritability, anorexia and vomiting were also encountered. In all cases a palpable abdominal mass was found and intravenous pyelography indicated this to be renal in origin. At laparotomy the tumours were regarded as nephroblastomata and nephrectomy was carried out. Case 2 had been treated for Hirschsprung's disease earlier in childhood.

DISCUSSION

The microscopic appearance in the tumours described in this report, although showing some variation, has a common pattern which is different from the nephroblastoma. The absence of metanephric differentiation is important and the scanty cuboidal-lined tubules at the periphery are not consistent with a diagnosis of Wilms' tumour. The predominant cell is polygonal with a pale, delicate-chromatin nucleus. Blastema is absent. Epithelial structures such as squamous elements and mesodermal tissue such as striated muscle, bone and cartilage have not been encountered. The features can be clearly distinguished from the nephroblastoma and, as stated in the introduction, the subsequent development of bone metastases was correctly predicted in several cases. The male sex preponderance, 13 boys and 2 girls, is different from expected (binomial test, $P<0{\cdot}01$). In Wilms' tumour the sex

FIG. 2.—Histological features of BMRTC. *Top left:* Predominant component with polygonal cells and prominent capillaries. Three tubules are present (H & E ×100). *Top right:* Collagenous area with a central tubule (H & E ×100). *Bottom left:* Fibrous bundles, isolated tubules and microcysts (H & E ×50). *Bottom right:* Loose fibroblastic area with liquefaction (H & E ×50).

TABLE.—*Clinical findings in* 15 *patients with bone-metastasizing renal tumour of childhood*

| Case and year of presentation | Age (yrs) | Sex | Side | Bone metastases | Onset of metastases | Progress (1978) |
|---|---|---|---|---|---|---|
| 1. C.N. (1957) | 4 | M | L | skull | 16 m. after presentation | Died $4\frac{1}{2}$ yr. Metastases in femur, humerus and skull |
| 2. D.G. (1968) | $1\frac{1}{2}$ | F | L | — | — | Died 18 days post-op. |
| 3. J.H. (1969) | $10\frac{1}{2}$ | M | R | — | — | Died post-op. |
| 4. M.F. (1970) | $1\frac{11}{12}$ | M | R | femur | 2 weeks after presentation | Died 6 m. "widespread metastases" |
| 5. S.R. (1970) | $1\frac{11}{12}$ | M | R | zygoma & femur | 7 m. after presentation | Died 13 m. Metastases in skull, scapulae, humeri, ribs, sternum, clavicle, L. tibia, vertebrae, pelvis, femora and ulna |
| 6. A.E.S. (1972) | $2\frac{9}{12}$ | M | L | rib | 18 m. aftei presentation | Alive. No further metastases |
| 7. T.M. (1972) | $4\frac{9}{12}$ | M | L | widespread | 21 m. after presentation | Died 22 m. Disseminated metastases to lung and bone |
| 8. G.B. (1973) | $4\frac{2}{12}$ | M | L | — | — | Died 2 days post-op. |
| 9. K.B.M. (1975) | 5 | M | L | rib | At presentation | Alive. (No further metastases) |
| 10. M.G.C. (1975) | $1\frac{5}{12}$ | M | L | calcaneum | 2 yr. after presentation | Alive. (No further metastases) |
| 11. D.S. (1975) | $6\frac{7}{12}$ | M | R | — | — | Alive. |
| 12. S.S.S. (1976) | $1\frac{1}{2}$ | M | L | clavicle | 8 m. after presentation | Developed metastases in skull and femur. Alive. |
| 13. J.W. (1976) | $1\frac{2}{12}$ | F | L | spine | 17 m. after presentation | Alive. (No further metastases) |
| 14. A.B. (1977) | $2\frac{10}{12}$ | M | R | — | — | Alive. |
| 15. P.T. (1977) | $2\frac{1}{2}$ | M | R | — | — | Alive. |

incidence is equal. However, the numbers in the present series are small and a larger group of cases will be required to establish this aspect.

The ages of the patients range between $1\frac{1}{2}$ and $10\frac{1}{2}$ years although there is only one case over 7 years. The largest group is in the 1–2-year-old period in which there are 5 cases and a smaller peak of 3 patients is seen in the age group 4–5 years.

The most important clinical feature is the high incidence of bone metastases which was seen in 9/15 patients. Osseous deposits were noted at presentation in one case, but did not develop until 2 years after nephrectomy in another patient. Three of the children died in the early postoperative period and 2 of the other 3 cases without bone metastases were diagnosed in 1977. It is possible that the incidence of bone metastases may be higher than the 60% found in this series at the present time.

The total number of primary renal tumours in this report which were initially thought to be Wilms' tumour is 358 and in the true nephroblastomata only 2 out of the 343 patients developed bone metastases. In one of these cases the tumour was a typical nephroblastoma with blastemal islands and metanephric tubular differentiation, while the other had collections of bizarre eosinophilic cells having a rhabdomyosarcomatous appearance. The latter patient died with spinal metastasis but necropsy permission was not obtained and it is not possible to know the histological appearance of the metastasis.

The incidence of bone metastases in Wilms' tumour is in the region of 3·5% (Bond & Martin, 1975). The incidence of the bone-metastasizing tumour described in this report in relation to primary childhood renal tumours is approximately 4% and from an analysis of the material in the present paper the incidence of bone metastases in true nephroblastoma is approximately 0·5%.

The histogenesis of the particular bone-metastasizing tumour is not known and requires further study. Kidd (1970) reported a bone-metastasizing sarcoma of the kidney in childhood which was distinct from nephroblastoma. An origin from mesangial or interstitial cells has been considered but, in the present state of know ledge, the term "bone-metastasizing renal tumour of childhood" is suggested for this entity.

### REFERENCES

BOND, J. V. & MARTIN, E. C. (1975) Bone metastases in Wilms' tumour. *Clin. Radiol.*, **26**, 103.

KIDD, J. M. (1970) Exclusion of certain renal neoplasms from the category of Wilms' tumour. *Am. J. Path.*, **58**, 16a.

MARSDEN, H. B., LAWLER, W. & KUMAR, P. (1978) Bone-metastasizing renal tumour of childhood. *Cancer* (in press) (October).